# Calycosin Attenuates LPS-Induced Cardiomyocyte Injury Through Regulation of the PPARγ/NF-κB Signaling Pathway

**DOI:** 10.3390/ijms27198505

**Published:** 2026-09-23

**Authors:** Rui Zhao, Zhiwang Wang, Wuzhou Liu, Keke Liang

**Affiliations:** College of Pharmacy, Gansu University of Chinese Medicine, Lanzhou 730000, China; 18709486574@163.com (R.Z.); 17789619085@163.com (W.L.)

**Keywords:** calycosin, cardiomyocyte injury, PPARγ signalling network, inflammation, oxidative stress

## Abstract

Calycosin (CAL) is an isoflavone monomer derived from *Astragalus membranaceus* that possesses potent anti-inflammatory and antioxidative pharmacological activities. However, the specific role of CAL in lipopolysaccharide (LPS)-induced inflammatory injury in cardiomyocytes remains to be elucidated. In this study, we established an in vitro inflammatory injury model using LPS-stimulated H9c2 cardiomyocytes to evaluate the cardioprotective effects of CAL. We assessed cell viability, live/dead cell staining, oxidative stress markers, inflammatory cytokine profiles, and cardiac injury biomarkers to characterize the protective efficacy of CAL. Quantitative proteomics combined with bioinformatics analysis was employed to explore the underlying mechanisms. Molecular docking and molecular dynamics simulations were performed to analyze the predicted binding affinity and complex stability between CAL and the target proteins PPARγ and NF-κB. PPARγ and NF-κB mRNA and protein levels were measured by qPCR and Western blotting, and rescue experiments with the PPARγ-specific inhibitor GW9662 were performed to validate target dependency. Our results demonstrated that CAL exerted significant protective effects against LPS-induced cardiomyocyte injury, effectively enhancing cell viability, reducing cell death, alleviating intracellular ROS accumulation, modulating oxidative stress-related parameters, suppressing excessive inflammatory responses, and decreasing the release of cardiac injury biomarkers. Proteomic analysis revealed that differentially expressed proteins were significantly enriched in the PPAR signaling pathway. Molecular simulations predicted that CAL binds stably to both PPARγ and NF-κB. PCR and Western blotting results showed that CAL upregulated PPARγ and suppressed NF-κB-mediated pro-inflammatory gene expression at both the mRNA and protein levels. Notably, pharmacological blockade of PPARγ with GW9662 partially abolished the cardioprotective effects of CAL. Collectively, these findings indicate that CAL ameliorates LPS-induced inflammatory injury in H9c2 cardiomyocytes through a protective mechanism that depends on activation of PPARγ and subsequent inhibition of the NF-κB signaling pathway.

## 1. Introduction

Excessive inflammation and oxidative stress injury in cardiomyocytes constitute a common pathological basis for the initiation and progression of various cardiovascular diseases. Lipopolysaccharide (LPS) can act directly on cardiomyocytes, triggering intracellular inflammatory cascades, inducing the release of abundant pro-inflammatory mediators, and causing burst-like accumulation of reactive oxygen species (ROS), which consequently leads to reduced cell viability, increased cell death, and elevated release of cardiac injury biomarkers [1,2]. Therefore, suppressing excessive inflammatory responses and ameliorating oxidative stress injury in cardiomyocytes are of great significance for cardioprotection. Currently, safe and highly effective targeted interventions for myocardial inflammatory injury remain scarce, and screening for cardioprotective candidate compounds from bioactive ingredients of traditional Chinese medicines represents a promising research avenue.

Calycosin (CAL) is a major isoflavone bioactive monomer derived from the traditional Chinese herb *Astragalus membranaceus*. Modern pharmacological studies have demonstrated that CAL possesses multiple biological activities, including anti-inflammatory, antioxidative and anti-cytotoxic effects, and exhibits notable cytoprotective efficacy in various myocardial injury models, such as doxorubicin-induced cardiotoxicity and myocardial ischemia–reperfusion injury [3,4]. Previous reports have indicated that CAL can inhibit multiple inflammatory signaling pathways, including the NF-κB pathway, and attenuate tissue inflammatory infiltration and oxidative damage [5]. Peroxisome proliferator-activated receptor gamma (PPARγ), a member of the nuclear receptor family, negatively regulates NF-κB-mediated inflammatory transcriptional programs and serves as a key molecular hub linking oxidative stress and inflammatory responses [6]. Prior studies in kidney injury models have suggested that CAL can upregulate PPARγ expression and consequently suppress downstream NF-κB pathway activity [7]. However, direct experimental evidence is still lacking as to whether CAL exerts its protective effects through modulation of the PPARγ/NF-κB axis in the LPS-induced inflammatory injury model of H9c2 cardiomyocytes.

In this study, we established an in vitro model of cardiomyocyte inflammatory injury using LPS-stimulated rat H9c2 cardiomyocytes, and examined the effects of CAL on cell viability, cell death, intracellular ROS levels, inflammatory cytokines, oxidative stress parameters and cardiac injury biomarkers. We further employed proteomic profiling to identify differentially expressed proteins and performed inhibitor-based rescue experiments (Figure S1). Collectively, this work aims to elucidate the molecular mechanisms underlying the cardioprotective action of CAL, and to provide in vitro experimental evidence for the development of candidate therapeutics against myocardial inflammatory injury.

## 2. Results

### 2.1. CAL Ameliorates LPS-Induced H9c2 Cell Injury and Suppresses Intracellular Reactive Oxygen Species Production

To determine the safe dosage range of CAL, we first evaluated the effect of varying concentrations of CAL on cell viability using the CCK-8 assay (Figure 1A). As shown in Figure 1B, treatment with CAL at concentrations ranging from 12.5 to 100 μmol/L did not significantly alter cell viability, indicating no apparent cytotoxicity within this concentration range. Following LPS stimulation to establish the cellular injury model, the Model group exhibited a marked decrease in cell viability (*p* < 0.01). Notably, intervention with CAL at different concentrations restored cell viability in a dose-dependent manner, with the most pronounced effects observed at 12.5, 25, and 50 μmol/L (*p* < 0.01) (Figure 1C). Accordingly, these three concentrations were selected for subsequent experiments and designated as CAL-low dose (CAL-L, 12.5 μmol/L), CAL-medium dose (CAL-M, 25 μmol/L), and CAL-high dose (CAL-H, 50 μmol/L).

Calcein-AM/PI live/dead cell staining revealed that the Ctrol group displayed abundant green-fluorescent viable cells with minimal PI-positive dead cells (Figure 1D). In contrast, the LPS-challenged Model group exhibited a substantial increase in PI-labeled dead cells. Remarkably, CAL treatment significantly reduced the number of dead cells compared with the Model group (*p* < 0.01) (Figure 1G). DHE staining was employed to evaluate intracellular superoxide anion levels (Figure 1E), while DCFH-DA staining was used to detect total intracellular ROS levels (Figure 1F). The LPS-treated Model group showed markedly enhanced DHE and DCFH-DA fluorescent signals, indicating that LPS stimulation caused massive accumulation of intracellular ROS. However, following CAL treatment at all three doses, the fluorescence intensities of both superoxide anions (Figure 1H) and total ROS (Figure 1I) were significantly reduced (*p* < 0.05, *p* < 0.01). Collectively, these findings demonstrate that CAL reverses LPS-induced loss of cell viability, reduces cell death, and suppresses LPS-mediated excessive intracellular ROS production, thereby exerting cytoprotective effects.

### 2.2. CAL Suppresses LPS-Induced Inflammatory Cytokine Release and Ameliorates Oxidative Stress in H9c2 Cells

Inflammatory response assays revealed that, compared with the Ctrol group, the Model group exhibited significantly elevated levels of NF-κB, IL-1β, IL-6, TNF-α and NLRP3 in the cell culture supernatant (*p* < 0.01), accompanied by a marked decrease in the anti-inflammatory cytokine IL-10 (*p* < 0.01) (Figure 2A–F). Notably, treatment with CAL at varying concentrations dose-dependently reduced the levels of NF-κB, IL-1β, IL-6, TNF-α and NLRP3, while concurrently upregulating the secretion of the anti-inflammatory factor IL-10 (*p* < 0.05, *p* < 0.01).

Assessment of oxidative stress parameters demonstrated that the Model group had significantly reduced levels of GSH and SOD, along with a marked increase in the lipid peroxidation product MDA (Figure 2G–I), indicating that LPS stimulation compromised cellular antioxidant capacity and exacerbated oxidative injury. Encouragingly, CAL treatment at all tested doses effectively elevated GSH and SOD levels, reduced MDA content, and ameliorated the oxidative stress status of the cells (*p* < 0.05, *p* < 0.01).

### 2.3. CAL Downregulates LPS-Induced Levels of Cardiac Injury Biomarkers

To evaluate the protective effect of CAL against LPS-induced cardiomyocyte injury, we measured the cardiac enzyme profile and cardiac-specific injury biomarkers. As shown in Figure 3A–F, compared with the Ctrol group, the Model group exhibited significantly elevated levels of the cardiac enzymes CK, CK-MB, HBDH and LDH, along with upregulated expression of the cardiac injury biomarkers MYO/MB and cTn-I (*p* < 0.05, *p* < 0.01), indicating that LPS stimulation caused substantial cardiomyocyte damage. Notably, CAL treatment at low, medium and high doses dose-dependently reduced the enzymatic activities of CK, CK-MB, HBDH and LDH, and downregulated the levels of MYO/MB and cTn-I compared with the Model group (*p* < 0.05, *p* < 0.01). Collectively, these findings further confirm that CAL attenuates the release of cardiac injury biomarkers and alleviates LPS-induced cardiomyocyte injury.

### 2.4. Proteomic Profiling Analysis

To further delineate the core regulatory mechanisms underlying CAL-mediated intervention in LPS-induced cardiomyocyte injury, we performed quantitative proteomic profiling to validate and extend the understanding of CAL’s targets and regulatory networks at the protein level. A Venn diagram summarizing protein expression across groups identified a total of 2713 commonly expressed proteins (Figure 4A). Principal component analysis (PCA) and hierarchical clustering heatmaps revealed a clear separation between the Ctrol and Model groups, confirming successful establishment of the cardiomyocyte injury model. Notably, CAL intervention partially reversed the protein expression profile of the Model group, demonstrating a definite modulatory effect of CAL on cardiomyocyte injury (Figure 4B–D). Differential protein expression analysis identified 81 differentially expressed proteins (DEPs) between the Model and Ctrol groups, of which 43 were upregulated and 38 downregulated (Figure 4E). Comparing the CAL-treated group with the Model group, 44 DEPs were identified, including 23 upregulated and 21 downregulated proteins (Figure 4F).

GO functional enrichment analysis of the DEPs revealed that biological processes (BP) were primarily involved in cell proliferation, inflammatory responses and oxidative stress; cellular components (CC) were mainly enriched in key structures such as the plasma membrane and cytoplasm; and molecular functions (MF) encompassed regulatory activities including protein binding and kinase activity, providing a functional foundation for subsequent pathway analysis (Figure 5A). KEGG pathway enrichment analysis demonstrated that DEPs associated with CAL intervention in cardiomyocyte injury were predominantly enriched in the PPAR signaling pathway, suggesting that the PPAR signaling pathway may serve as the core regulatory axis mediating the therapeutic effects of CAL in cardiomyocyte injury (Figure 5B–D).

### 2.5. Effects of CAL on the PPARγ/NF-κB Signaling Pathway in H9c2 Cells

To explore the potential molecular targets through which CAL regulates cardiomyocyte injury, we performed molecular docking analysis. The results revealed that CAL exhibited favorable binding affinities for both PPARγ and NF-κB, with binding energies of −9.0 kcal/mol and −6.9 kcal/mol, respectively (Figure 6A,B). To further evaluate the interaction between CAL and PPARγ and the stability of the resulting complex, MD simulations were conducted. Three independent MD systems were simulated: PPARγ alone, CAL alone, and the PPAR-CAL complex. As shown in Figure 6C, the RMSD of PPARγ alone reached a plateau at approximately 0.27 nm, the RMSD of CAL alone remained at approximately 0.04 nm, and the RMSD of the PPARγ–CAL complex stabilized at approximately 0.25 nm in the later stage of the simulation, indicating that all three systems adopted stable conformations. The Rg and SASA of the complex remained stable at approximately 1.93 nm and 148 nm^2^, respectively (Figure 6D,E), indicating that the complex retained a compact structure. Hydrogen-bond analysis showed that 2–4 hydrogen bonds were persistently formed between CAL and PPARγ (Figure 6F). RMSF analysis showed that the Cα atoms of PPARγ fluctuated mainly in the range of 0.05–0.35 nm (Figure 6G). The FEL analysis identified a global minimum at index 589 (energy = 0.000) and a secondary minimum at index 587 (energy = 0.107), indicating that the PPARγ–CAL complex adopted a stable low-free-energy conformation (Figure 6H). These results should be interpreted as computational predictions from single 100 ns trajectories and do not constitute experimental evidence of binding.

RT-qPCR results showed that, compared with the Ctrol group, the Model group exhibited significantly downregulated PPARγ mRNA expression, accompanied by markedly upregulated mRNA levels of NF-κB and TNF-α in H9c2 cells. Treatment with CAL at varying concentrations (12.5, 25, and 50 μmol/L) effectively upregulated PPARγ mRNA expression, while concurrently reducing the mRNA expression levels of NF-κB and its downstream inflammatory cytokine TNF-α (Figure 6I–K). To validate this finding at the protein level, we performed Western blotting and confirmed that CAL activates the PPAR signaling pathway and inhibits NF-κB expressions (Figure 6L).

### 2.6. GW9662 Abrogates CAL-Mediated Regulation of the PPAR/NF-κB Signaling Pathway

To validate the pivotal role of the PPAR signaling pathway in calycosin (CAL)-mediated cytoprotection, we performed rescue experiments using the PPAR-specific inhibitor GW9662 (Figure 7A). CCK-8 assay results demonstrated that CAL significantly reversed LPS-induced reduction in cell viability; however, upon inhibition of PPARγ activity with GW9662, the cytoprotective effect of CAL was partially counteracted, as evidenced by a decline in cell viability compared with the CAL-alone group (Figure 7B). Calcein-AM/PI live/dead cell staining revealed that CAL treatment markedly reduced the number of PI-positive dead cells induced by LPS; conversely, co-incubation with GW9662 resulted in a re-increase in dead cell numbers, indicating that the anti-cytotoxic effect of CAL was attenuated (Figure 7C,E). DHE staining showed that CAL effectively reduced LPS-elevated superoxide anion levels; notably, this ROS-scavenging effect of CAL was significantly blunted when PPARγ was blocked by GW9662 (Figure 7D,F).

To further investigate the antagonistic effect of GW9662 on CAL-mediated protection, we measured cardiac injury biomarkers and the mRNA expression levels of pathway-related genes. As shown in Figure 8A–F, co-treatment with CAL and GW9662 significantly reversed the protective effects of CAL on cardiac injury biomarkers, with these injury-related parameters rebounding compared with the CAL-alone group. qPCR analysis revealed that PPARγ inhibition by GW9662 abolished the CAL-induced upregulation of PPARγ mRNA, and concurrently counteracted the transcriptional repression of NF-κB and TNF-α by CAL (Figure 8G–I). Collectively, these results confirm that the protective effect of CAL against LPS-induced cardiomyocyte injury is dependent on activation of the PPARγ pathway, which in turn suppresses downstream NF-κB-mediated inflammatory responses.

## 3. Discussion

Myocardial injury represents a common pathological substrate underlying the initiation and progression of various cardiovascular diseases, with its detrimental impact spanning a broad clinical spectrum ranging from acute critical illnesses to chronic degenerative conditions. In the context of sepsis, approximately 40–50% of patients develop septic cardiomyopathy, and the mortality rate among these patients is significantly higher than that of those without cardiac involvement [8]. Cardiomyocytes, as terminally differentiated cells, possess extremely limited regenerative capacity; once extensive injury occurs, irreversible cardiac dysfunction ensues, ultimately culminating in heart failure. In high-altitude environments, factors such as hypoxia compounded with infection can induce excessive inflammatory responses and oxidative stress injury in cardiomyocytes, leading to high-altitude cardiac injury that poses a serious threat to the safety of individuals rapidly ascending to high-altitude regions [9]. Despite considerable advances in the diagnosis and treatment of cardiovascular diseases in modern medicine, safe and highly effective targeted interventions for the aforementioned myocardial inflammatory injury remain scarce. Consequently, screening for cardioprotective candidate compounds from natural products has emerged as a critical direction in cardiovascular pharmacological research.

Calycosin (CAL) is a major isoflavone bioactive monomer derived from *Astragalus membranaceus*. Modern pharmacological studies have established that CAL possesses multiple biological activities, including anti-inflammatory, antioxidative, anti-apoptotic, immunomodulatory and cardiovascular protective effects. In myocardial ischemia–reperfusion injury models, CAL has been shown to reduce myocardial infarct size and improve cardiac function [10]. In doxorubicin-induced cardiotoxicity models, CAL attenuates oxidative stress and inflammatory injury through regulation of the Sirt1-NLRP3 pathway [11]. However, direct experimental evidence has been lacking regarding whether CAL exerts its protective effects via modulation of the PPARγ/NF-κB signaling axis in the LPS-induced cardiomyocyte inflammatory injury model, a classical in vitro system that recapitulates septic cardiomyopathy and infection-related myocardial injury. To address this gap, the present study employed rat H9c2 cardiomyocytes and integrated pharmacological intervention, proteomic screening and molecular biological validation to systematically investigate the protective effects and underlying mechanisms of CAL against LPS-induced cardiomyocyte injury.

Excessive activation of inflammatory responses and oxidative stress constitutes the two core driving factors in LPS-induced cardiomyocyte injury. Our results demonstrated that CAL significantly reduced the levels of pro-inflammatory factors, including NF-κB, IL-1β, IL-6, TNF-α and NLRP3, in the cell culture supernatant following LPS stimulation, while concurrently upregulating the secretion of the anti-inflammatory cytokine IL-10. Wang et al. [12], in a rat model of chronic prostatitis, similarly found that CAL significantly decreased serum and prostatic tissue levels of IL-1β, IL-6 and TNF-α, and alleviated tissue inflammatory injury through inhibition of the p38 MAPK/NF-κB pathway. Using DHE and DCFH-DA fluorescent probes, we observed that CAL markedly reduced LPS-induced intracellular superoxide anion and total ROS levels, restored the activities of the antioxidant enzymes SOD and GSH, and decreased the content of the lipid peroxidation product MDA. Liu et al. [13], using a rat model of septic myocardial injury, found that CAL inhibited myocardial inflammation and oxidative stress through regulation of the TLR4/p38 MAPK pathway. The cardioprotective effect of CAL was further corroborated by measurements of cardiac enzymes and specific injury biomarkers. Following LPS stimulation, the activities of cardiac enzymes CK, CK-MB, HBDH and LDH, as well as the levels of the cardiac injury biomarkers MYO/MB and cTn-I, were significantly elevated, reflecting compromised cardiomyocyte membrane integrity and release of intracellular proteins [14]. CAL treatment dose-dependently reversed these alterations. cTn-I, considered the “gold standard” biomarker of myocardial injury, exhibits a high degree of correlation with the extent of cardiomyocyte necrosis [15]. The marked suppression of cTn-I release by CAL in our study strongly supports its direct cardiomyocyte-protective action. Zhu et al. [16] demonstrated that verbascoside reduced circulating CK-MB and LDH levels and improved left ventricular ejection fraction (EF) and fractional shortening (FS) in septic mice. The consistent ability of diverse natural phenolic compounds to attenuate cardiac enzyme abnormalities across different myocardial injury models suggests that suppression of inflammation-mediated cardiomyocyte membrane damage may represent a shared pharmacological feature of this class of compounds [17].

PPARs belong to subfamily 1 of the nuclear receptor superfamily and function as ligand-dependent transcriptional regulators. In cardiomyocytes, activation of PPARγ suppresses the expression of multiple pro-inflammatory factors, including TNF-α, IL-1β, IL-6, MCP-1 and cell adhesion molecules [18,19]. In LPS-induced septic myocardial injury models, the PPARγ agonist pioglitazone has been shown to significantly reduce myocardial NF-κB activity, decrease pro-inflammatory cytokine release and improve cardiac function [20]. In doxorubicin-induced cardiotoxicity models, PPARγ activation alleviates myocardial injury by suppressing oxidative stress and mitochondrial dysfunction [21]. Furthermore, PPARγ is also involved in the regulation of autophagy, apoptosis and fibrosis in cardiomyocytes. To further validate the causal role of this pathway in the protective effects of CAL, we employed the PPARγ-specific irreversible antagonist GW9662 in rescue experiments. Our results showed that GW9662 not only abolished CAL-induced upregulation of PPARγ mRNA expression, but also significantly antagonized its transcriptional repression of NF-κB and TNF-α. Concurrently, the protective effects of CAL in reducing cardiac injury biomarkers, decreasing cell death and clearing ROS were all partially reversed. Studies in renal ischemia–reperfusion injury models have further confirmed that CAL inhibits NF-κB-mediated inflammatory responses through the PPARγ/EGR1 pathway [22]. This study was conducted only at the in vitro cellular level. Although this cell line retains many key phenotypic characteristics of cardiomyocytes, the in vitro cell model cannot fully recapitulate the complex in vivo microenvironment, including the potential effects of pharmacokinetics, plasma protein binding, tissue perfusion heterogeneity, and neurohumoral modulation on the pharmacological actions of CAL. Therefore, further in vivo animal studies are warranted to confirm the protective effects of CAL against myocardial injury.

In summary, CAL exerts its cardiomyocyte-protective effects by activating PPARγ, which in turn suppresses the NF-κB signaling pathway, thereby attenuating inflammatory responses and oxidative stress injury (Figure 9).

## 4. Materials and Methods

### 4.1. Cells, Reagents, and Instrumentation

Rat H9c2 cardiomyocytes were purchased from Wuhan Procell Life Science & Technology Co., Ltd. (Wuhan, China). Calycosin (CAL) was obtained from Chengdu Nakeli Biotechnology Co., Ltd. (NK1-00176, Chengdu, China). Cell Counting Kit-8 (CCK-8) was purchased from TargetMol (C0005, Boston, MA, USA). The reactive oxygen species (ROS) assay kit (DCFH-DA, S0033), superoxide anion assay kit (DHE, S0064), lipopolysaccharide (LPS, ST1470) and GW9662 (PPAR antagonist, SC9123) were obtained from Beyotime Biotechnology (Shanghai, China). ELISA kits for TNF-α (MM-0180R1), IL-1β (MM-0047R1), IL-10 (MM-0195R2), IL-6 (MM-0190R1), SOD (MM-0386R1), MDA (MM-0385R1), GSH (MM-0602R1), MYO/MB (MM-0921H2) and cTn-I (MM-60103H2), as well as the BCA protein assay kit (MM-9227A), were purchased from Jiangsu Meimian Industrial Co., Ltd. (Xuzhou, Jiangsu, China). GAPDH (60004-1-Ig), p-NF-κB (82335-1-RR), and PPARγ (16643-1-AP) were purchased from Proteintech (Wuhan, China).

### 4.2. Cell Viability Assay

H9c2 cells in the logarithmic growth phase were seeded into 96-well plates at a density of 5000 cells/well. After 24 h of culture, cells were treated with CAL at final concentrations of 12.5, 25, 50 and 100 μmol/L (six replicate wells per group), with a drug-free blank control group included. Following 24 h of incubation, 10 μL of CCK-8 solution was added to each well, and the plates were incubated at 37 °C for 1–2 h. The optical density (OD) at 450 nm was measured using a microplate reader(Tecan, Männedorf, Switzerland) to evaluate the effect of CAL alone on cell viability.

Concentrations of CAL that exerted no significant effect on cell viability (cell viability ≥90%) were selected for subsequent experiments. H9c2 cells were seeded into 96-well plates at 5000 cells/well and cultured for 24 h. The following groups were established: blank control group, Model group (1 μg/mL LPS for 24 h), and CAL + Model groups (various concentrations of CAL for 2 h, followed by co-incubation with 1 μg/mL LPS for 24 h), with six replicate wells per group. After treatment, cell viability was assessed using the CCK-8 assay. The optimal concentrations that effectively ameliorated LPS-induced cellular injury were selected for subsequent mechanistic studies.

### 4.3. Calcein-AM/PI Staining Assay

Cell viability and death were assessed using Calcein-AM/PI dual fluorescence staining. H9c2 cells were seeded into confocal culture dishes and subjected to the designated treatments. After treatment, the culture medium was discarded, and cells were washed twice with PBS. A staining solution containing 2 μmol/L Calcein-AM and 4 μmol/L PI was added, and cells were incubated at 37 °C for 20 min in the dark. After removing the staining solution and washing with PBS, images were immediately captured under a fluorescence microscope (Olympus, Tokyo, Japan) (Calcein-AM: excitation/emission = 488/515 nm, green fluorescence, representing live cells; PI: excitation/emission = 535/617 nm, red fluorescence, representing dead cells). Relative red fluorescence intensity (normalized to the blank control group) was quantified using ImageJ software(version 1.54f).

### 4.4. Measurement of Intracellular Total ROS Levels

Intracellular total ROS levels were measured using the ROS fluorescent probe DCFH-DA. H9c2 cells were seeded into 6-well plates and subjected to CAL and LPS treatments. After treatment, the culture medium was discarded, and cells were incubated with DCFH-DA probe (final concentration 10 μmol/L) diluted in serum-free medium at 37 °C for 30 min in the dark. Cells were washed three times with serum-free medium to remove unbound probe. Fluorescence intensity was measured using a fluorescence microplate reader(Tecan, Männedorf, Switzerland) at excitation/emission wavelengths of 488/525 nm. Results were expressed as relative fluorescence intensity (normalized to the blank control group).

### 4.5. Measurement of Intracellular Superoxide Anion Levels

Intracellular superoxide anion (O_2_^−^) levels were measured using the superoxide-specific fluorescent probe dihydroethidium (DHE). Following cell seeding and treatment, cells were incubated with DHE probe diluted in serum-free medium at 37 °C for 30 min in the dark. After washing with PBS, fluorescence intensity was immediately measured using a fluorescence microplate reader at excitation/emission wavelengths of 518/605 nm.

### 4.6. Cytokine and Biochemical Marker Measurements

Culture supernatants were collected after the completion of interventions and centrifuged at 3500× *g* for 15 min at 4 °C. The supernatants were aliquoted and stored at −80 °C for further analysis. Levels of NF-κB, interleukin-1β (IL-1β), IL-6, IL-10, tumor necrosis factor-α (TNF-α), NLRP3, superoxide dismutase (SOD), glutathione peroxidase (GSH-Px) activity and malondialdehyde (MDA) content in the supernatant were measured using commercial enzyme-linked immunosorbent assay (ELISA) kits strictly according to the manufacturers’ instructions. Additionally, cell lysates were collected for detection of MYO/MB and cTn-I expression.

### 4.7. Cardiac Enzyme Profile Measurement

Culture supernatants were collected after the designated interventions and centrifuged at 3500× *g* for 15 min at 4 °C to remove cells and debris. The cleared supernatants were then analyzed using a fully automatic biochemical analyzer (Roche cobas c 311, Roche Diagnostics, Shanghai, China) to measure the enzymatic activities of creatine kinase (CK), creatine kinase-MB isoenzyme (CK-MB), lactate dehydrogenase (LDH) and α-hydroxybutyrate dehydrogenase (HBDH), strictly in accordance with the manufacturer’s instructions.

### 4.8. Inhibitor Rescue Experiments

To investigate whether CAL exerts its cardioprotective effects through activation of PPARγ, the PPARγ-specific irreversible antagonist GW9662 was used for pathway validation. Based on literature reports and preliminary experimental results, the optimal working concentration of GW9662 was determined to be 10 μmol/L. H9c2 cells were seeded into 6-well plates and, after adherence, randomly divided into five groups: Ctrol group, Model group (1 μg/mL LPS for 24 h), CAL group (50 μmol/L), GW9662 group (10 μmol/L), and CAL + GW9662 group. Following the designated interventions, cells and culture supernatants were collected for measurement of the aforementioned parameters to determine whether GW9662 reversed the protective effects of CAL.

### 4.9. Proteomic Profiling

#### 4.9.1. Cell Sample Preparation

H9c2 cells were seeded into 10 cm culture dishes at an appropriate density. When cells reached approximately 80% confluence, they were divided into the following three groups: Ctrol group, Model group (1 μg/mL LPS for 24 h), and CAL group (50 μmol/L CAL for 2 h, followed by co-incubation with 1 μg/mL LPS for 24 h). After treatment, the culture medium was discarded, and cells were washed three times with ice-cold PBS. RIPA lysis buffer containing protease and phosphatase inhibitors was added, and cells were lysed on ice for 30 min. Cell lysates were collected using a cell scraper and centrifuged at 12,000× *g* for 20 min at 4 °C. The supernatants were collected, and protein concentrations were determined using the BCA method. Equal amounts of protein (≥100 μg) from each group were taken for subsequent enzymatic digestion.

#### 4.9.2. Protein Digestion and Peptide Purification

Protein solutions from each sample were reduced by adding dithiothreitol (DTT) to a final concentration of 10 mmol/L and incubating at 56 °C for 1 h, followed by alkylation with iodoacetamide (IAA) at a final concentration of 20 mmol/L for 30 min at room temperature in the dark. Pre-chilled acetone was added, and proteins were precipitated at −20 °C overnight. After centrifugation, the protein pellets were collected, resolubilized, and digested with trypsin (protein:enzyme ratio = 50:1) at 37 °C overnight. The resulting peptides were desalted and purified using C18 solid-phase extraction cartridges, vacuum-dried, and stored at −80 °C until further use.

#### 4.9.3. LC-MS/MS Analysis

Each sample was separated on a Vanquish neo nano-UPLC system coupled to an Astral mass spectrometer equipped with a nanospray ion source (Thermo Fisher Scientific, Waltham, MA, USA). Chromatographic separation was performed on a reversed-phase column (EASY-Spray™ HPLC, 150 μm × 15 cm, Thermo Fisher Scientific, USA). Mobile phase A was 0.1% formic acid in water and mobile phase B was 0.1% formic acid in 80% acetonitrile. The column was equilibrated with 96% A, and samples were loaded directly onto the column via an autosampler followed by gradient separation (Table S1). The separation gradient was applied according to the manufacturer’s recommended protocol for the EASY-Spray column.

Mass spectrometric data were acquired in data-independent acquisition (DIA) mode with positive ion detection. The full-scan (MS1) range was 380–980 *m*/*z* at a resolution of 240,000. The isolation window was 2 *m*/*z* and the normalized collision energy (NCE) was 25%. The MS2 scan range was 150–2000 *m*/*z*, with an absolute AGC value of 5E4 and a maximum injection time of 3 ms. Four biological replicates were analyzed per group.

#### 4.9.4. DIA Quantitative Analysis

Raw DIA files were processed with DIA-NN v1.8.1 in library-free mode against the Rattus norvegicus UniProt reference proteome (UP000002494). In this mode, the spectral library was generated in silico by DIA-NN using its deep learning-based prediction algorithm; no experimental DDA library was constructed and no peptide fractionation was performed prior to DIA. The false discovery rate (FDR) was controlled at <1% at both precursor and protein levels. Protein quantification was based on the MaxLFQ algorithm with sample-wise median normalization. Four biological replicates were analyzed per group. Missing values were handled by DIA-NN default processing, and only proteins quantified in at least 2 of 4 replicates of at least one group were retained for downstream analysis.

Differential expression was assessed by a two-group comparison based on the normalized MaxLFQ intensities. *p* values were computed by a moderated *t*-test as implemented in DIA-NN. Proteins were considered differentially expressed when |log_2_FC| > 1 and *p* < 0.05.

#### 4.9.5. GO and KEGG Enrichment Analysis

GO functional enrichment (biological process, cellular component, and molecular function) and KEGG pathway enrichment analyses were performed using Metascape (http://metascape.org, accessed on 10 March 2026). The organism was set to Rattus norvegicus. The background/reference protein set was the entire set of proteins quantified in this experiment. Upregulated and downregulated differentially expressed proteins were analyzed together as a single input list. Enrichment significance was assessed by accumulative hypergeometric distribution, and *p* values were adjusted for multiple testing using the Benjamini–Hochberg method. Terms with adjusted *p* < 0.05 were considered significantly enriched.

### 4.10. Molecular Docking

To predict the predicted binding affinity and interaction modes between CAL and PPARγ/NF-κB, molecular docking was performed. The three-dimensional structure of CAL was obtained from the PubChem database, and the crystal structures of PPARγ and NF-κB were downloaded from the RCSB Protein Data Bank (PDB). Using AutoDockTools 1.5.7, protein structures were pre-processed by removing water molecules, adding hydrogen atoms and calculating Gasteiger charges. The ligand CAL was prepared by defining rotatable bonds and assigning atom types. Semi-flexible docking was performed using AutoDock Vina 1.2.6 software. The grid box size and coordinates were determined based on literature reports and active-site predictions, with the exhaustiveness parameter set to 8. After docking, the optimal binding conformations were selected based on the lowest binding free energy (more negative values indicating higher predicted binding affinity). Visualization of docking poses was performed using PyMOL 2.5 (Table S2).

### 4.11. Molecular Dynamics Simulation

MD simulations were performed with GROMACS 2022.2 using the CHARMM36 force field. CAL was parameterized with CGenFF. Three independent systems were simulated: PPARγ alone, CAL alone, and the PPARγ–CAL complex. Each system was solvated in a cubic SPC/E water box with Na^+^/Cl^−^ counterions. After energy minimization, each system was equilibrated under NVT (310 K) and NPT (1 atm) ensembles, followed by a 100 ns unrestrained production run with a 2 fs time step. One 100 ns trajectory was performed for each system, and this is acknowledged as a limitation.

RMSD was calculated for the PPARγ backbone atoms, the CAL heavy atoms and all atoms of the PPARγ–CAL complex, each after least-squares fitting to the initial structure. RMSF was calculated for PPARγ Cα atoms. Rg and SASA were calculated for the PPARγ–CAL complex. Hydrogen bonds were defined by a donor–acceptor distance ≤3.5 Å and a donor–H–acceptor angle ≥120°. Free-energy landscapes (FELs) were constructed from the RMSD–Rg trajectories using gmx sham at 310 K (Table S3).

### 4.12. RT-qPCR

Total RNA was extracted from H9c2 cells in each group using TRIzol reagent. A total of 1 μg of RNA was reverse-transcribed into cDNA using a reverse transcription kit according to the manufacturer’s instructions. qPCR was performed using ChamQ Universal SYBR qPCR Master Mix on a LightCycler 480 II real-time PCR system. The 20 μL reaction mixture was subjected to the following cycling conditions: 95 °C for 30 s (pre-denaturation); 40 cycles of 95 °C for 10 s (denaturation), 60 °C for 30 s (annealing) and 72 °C for 30 s (extension). β-Actin was used as the internal reference gene, and relative gene expression was calculated using the 2^−ΔΔC^ method. All primers were designed and synthesized by Hebei Xiankang Biotechnology Co., Ltd. (Shijiazhuang, China). The primer sequences are listed in Table 1.

### 4.13. Statistical Analysis

All experimental data are presented as the mean ± standard deviation (SD). Comparisons among multiple groups were performed using one-way analysis of variance (ANOVA). When the overall difference reached statistical significance, post hoc pairwise comparisons were conducted using the least significant difference (LSD)-*t* test. A value of *p* < 0.05 was considered statistically significant. All statistical analyses and graphical representations were carried out using GraphPad Prism 8.3.0 software.

## Figures and Tables

**Figure 1:**
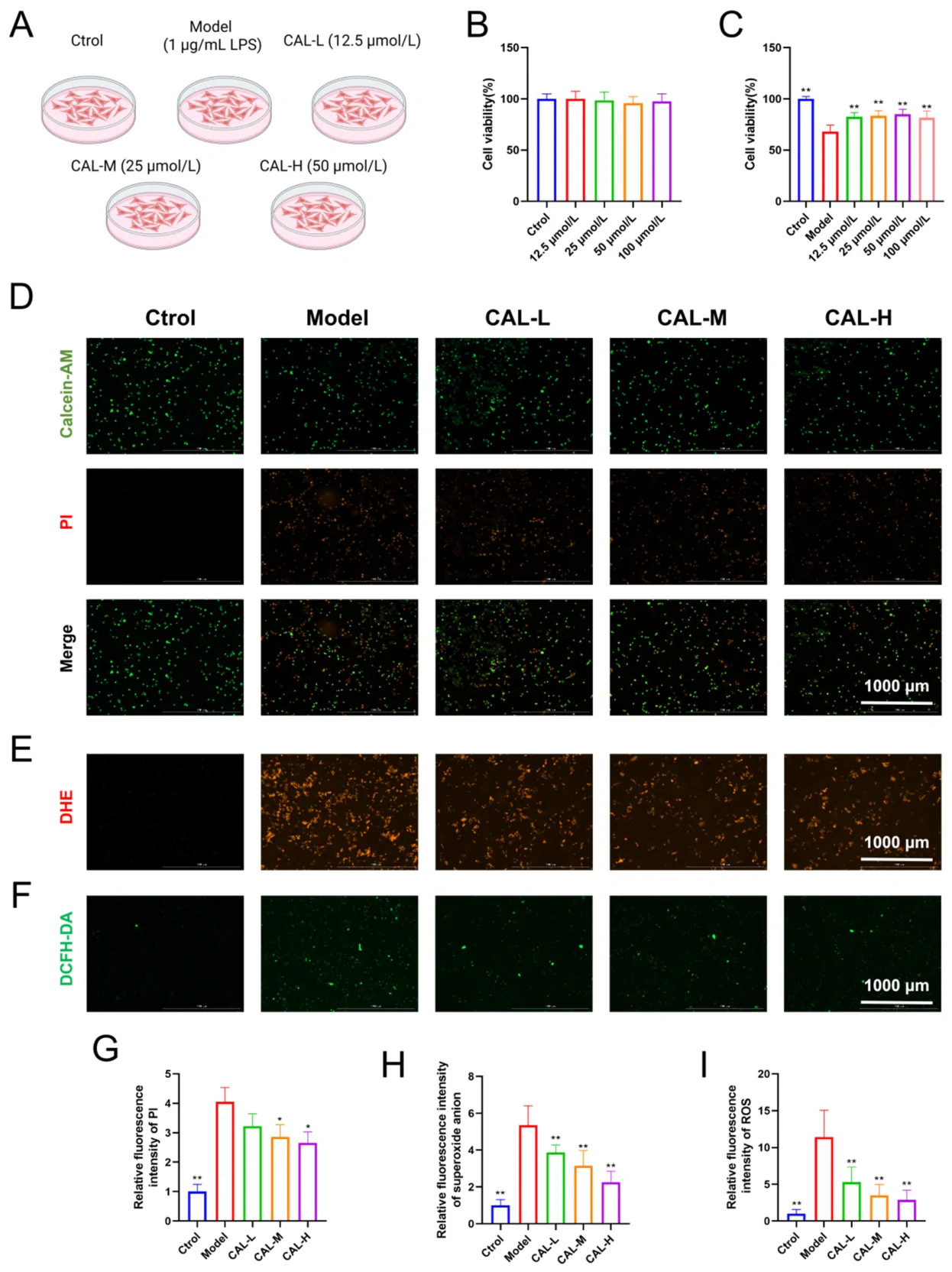
Effects of CAL on cell viability and intracellular reactive oxygen species levels in LPS-injured H9c2 cells. (**A**) Schematic diagram of experimental grouping and drug treatment regimen. (**B**,**C**) Cell viability assessed by CCK-8 assay. (**D**) Calcein-AM/PI staining for live/dead cell discrimination. (**E**) DHE staining for detection of intracellular superoxide anion levels. (**F**) DCFH-DA staining for detection of total ROS levels. (**G**) Relative fluorescence intensity of PI. (**H**) Relative fluorescence intensity of superoxide anion. (**I**) Relative fluorescence intensity of ROS. * *p* < 0.05 and ** *p* < 0.01 versus Model group. CAL-L: CAL-low dose; CAL-M: CAL-medium dose; CAL-H: CAL-high dose. Figure 1A was created with BioRender. Liang, K. (2026) https://BioRender.com/chm09me (accessed on 15 August 2026).

**Figure 2:**
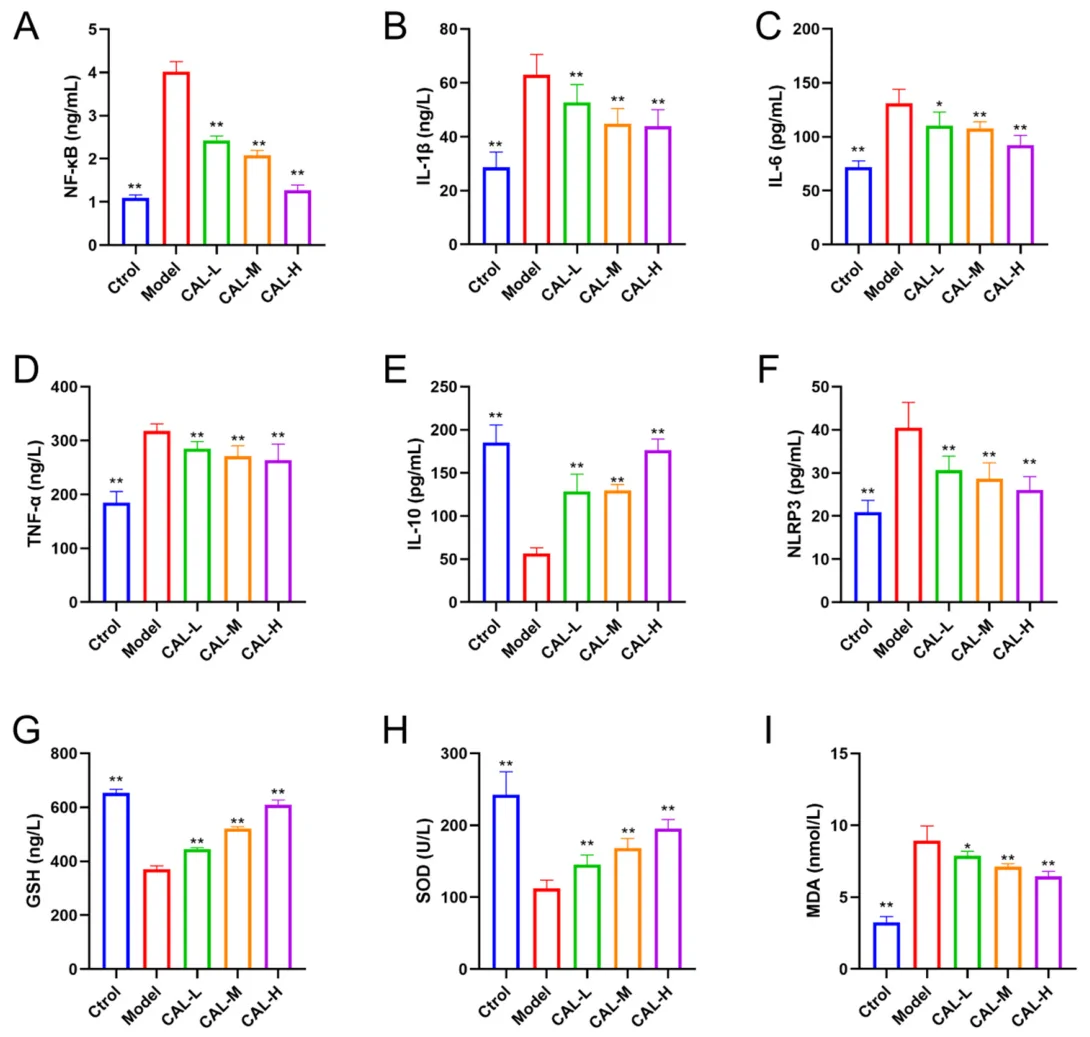
Effects of CAL on inflammatory and oxidative stress-related indicators in LPS-injured H9c2 cells. (**A**) NF-κB: nuclear factor-κB; (**B**) IL-1β: interleukin-1β; (**C**) IL-6: interleukin-6; (**D**) TNF-α: tumor necrosis factor-α; (**E**) IL-10: interleukin-10; (**F**) NLRP3: NOD-like receptor protein 3; (**G**) GSH: glutathione; (**H**) SOD: superoxide dismutase; (**I**) MDA: malondialdehyde. CAL-L: CAL-low dose; CAL-M: CAL-medium dose; CAL-H: CAL-high dose. * *p* < 0.05 and ** *p* < 0.01 compared with the Model group.

**Figure 3:**
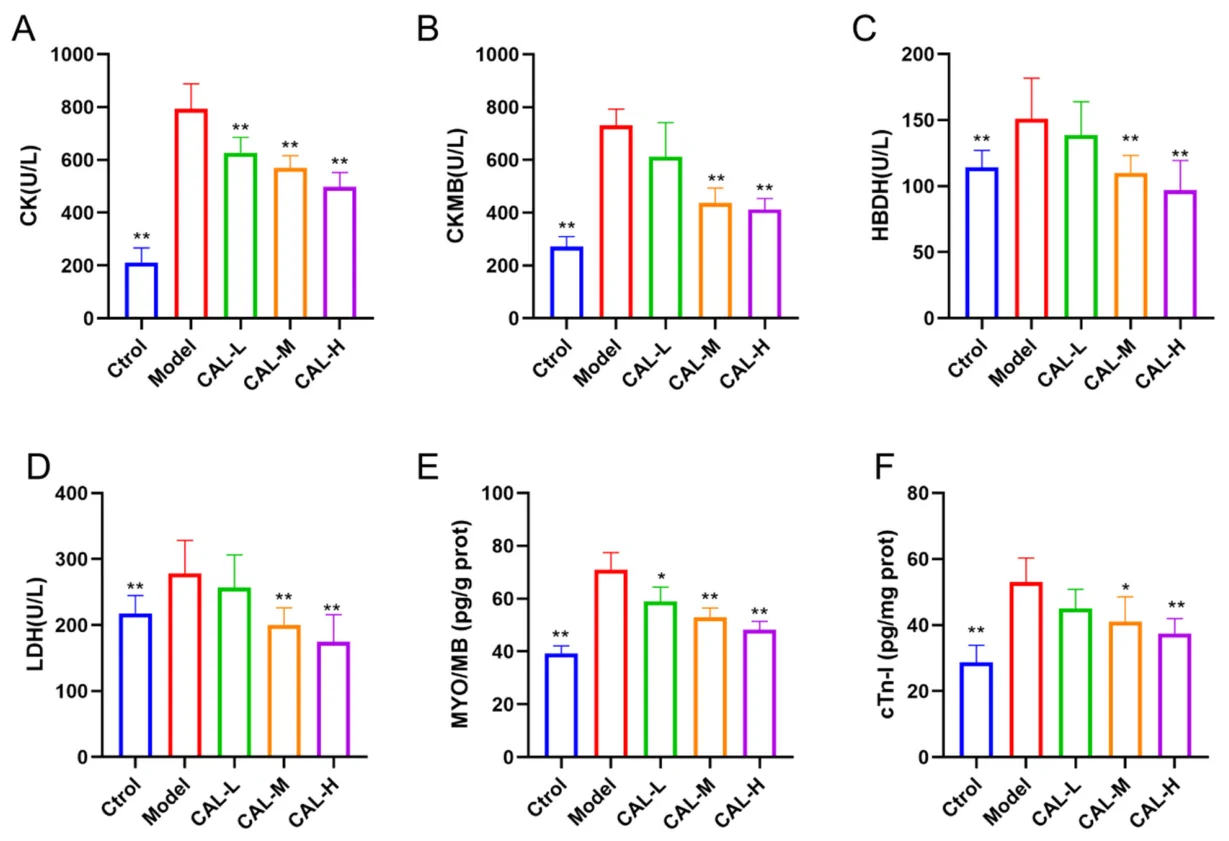
Effects of CAL on myocardial enzyme spectrum and myocardial injury markers under LPS-induced injury. (**A**) CK: creatine kinase; (**B**) CK-MB: creatine kinase-MB; (**C**) HBDH: α-hydroxybutyrate dehydrogenase; (**D**) LDH: lactate dehydrogenase; (**E**) MYO/MB: myoglobin; (**F**) cTn-I: cardiac troponin-I. CAL-L: CAL-low dose; CAL-M: CAL-medium dose; CAL-H: CAL-high dose. * *p* < 0.05 and ** *p* < 0.01 compared with the Model group.

**Figure 4:**
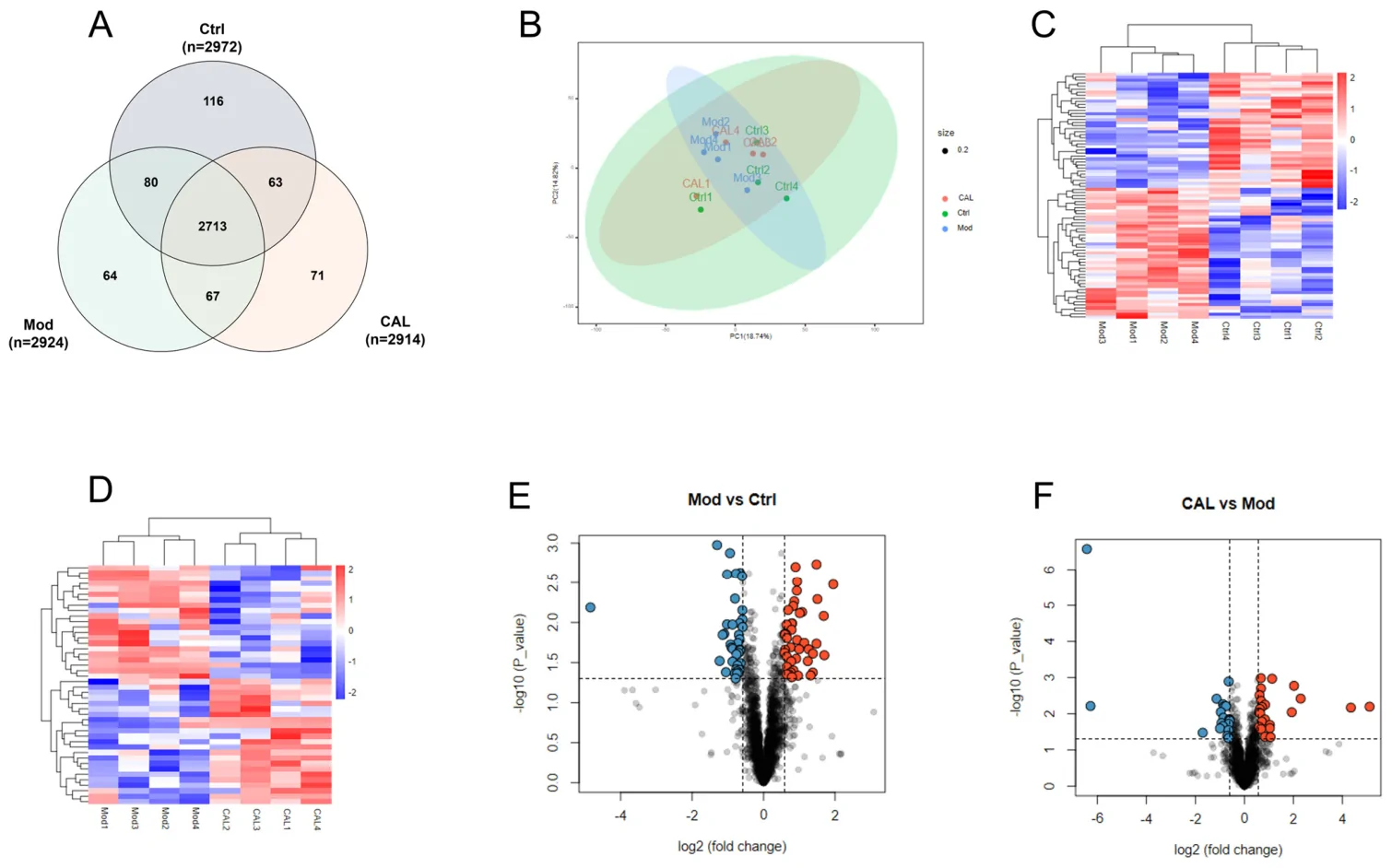
Proteomic profiling analysis of differentially expressed proteins. (**A**) Venn diagram of identified proteins; (**B**) principal component analysis (PCA); (**C**,**D**) hierarchical clustering heatmaps of differentially expressed proteins; (**E**) volcano plot of Mod vs. Ctrol comparison; (**F**) volcano plot of CAL vs. Mod comparison, Red dots indicate upregulated proteins; blue dots indicate downregulated proteins.

**Figure 5:**
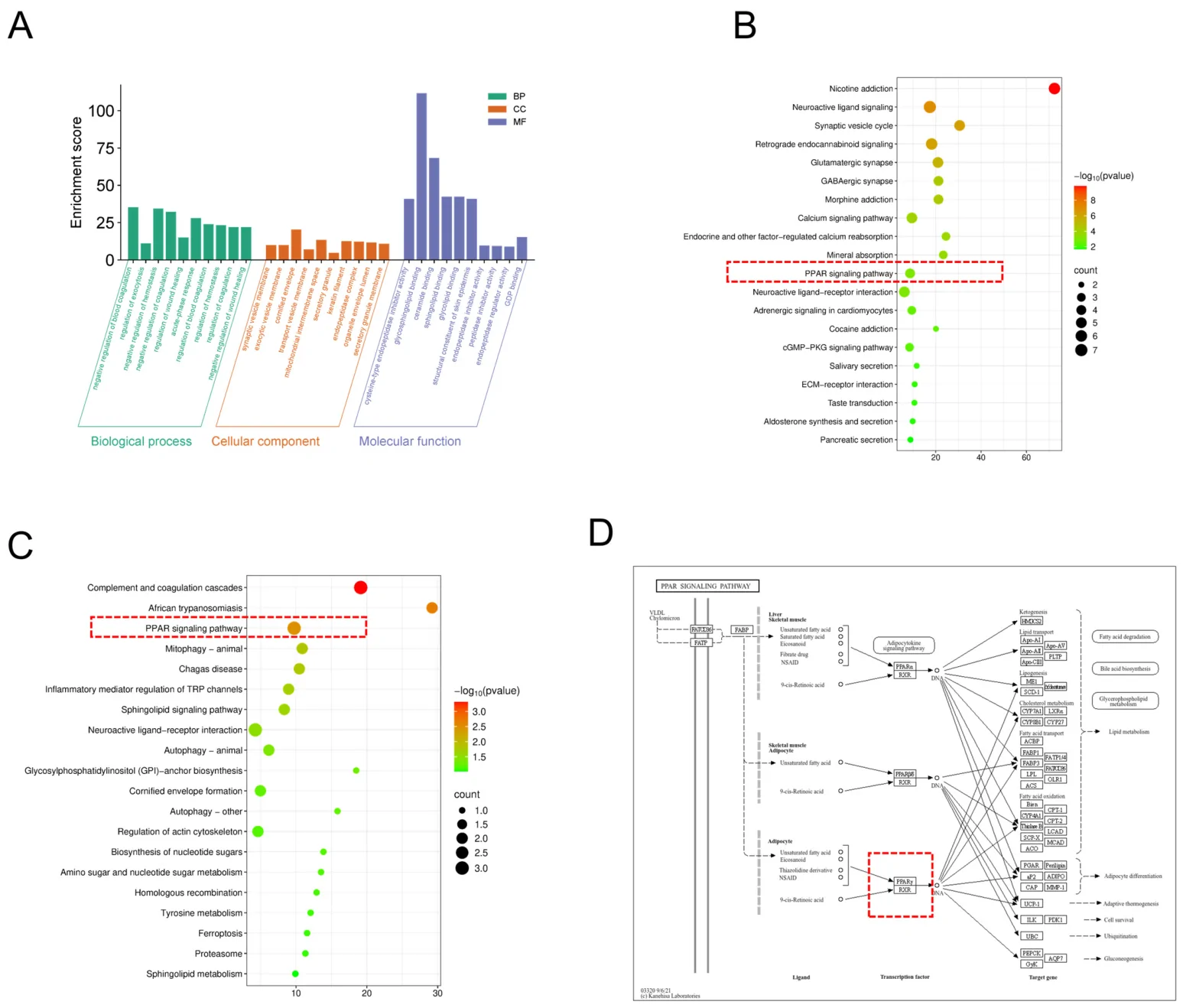
GO and KEGG enrichment analysis. (**A**) GO functional enrichment analysis of differential proteins, including biological process (BP), cellular component (CC), and molecular function (MF); (**B**,**C**) KEGG pathway enrichment bubble diagrams; (**D**) PPAR signaling pathway.

**Figure 6:**
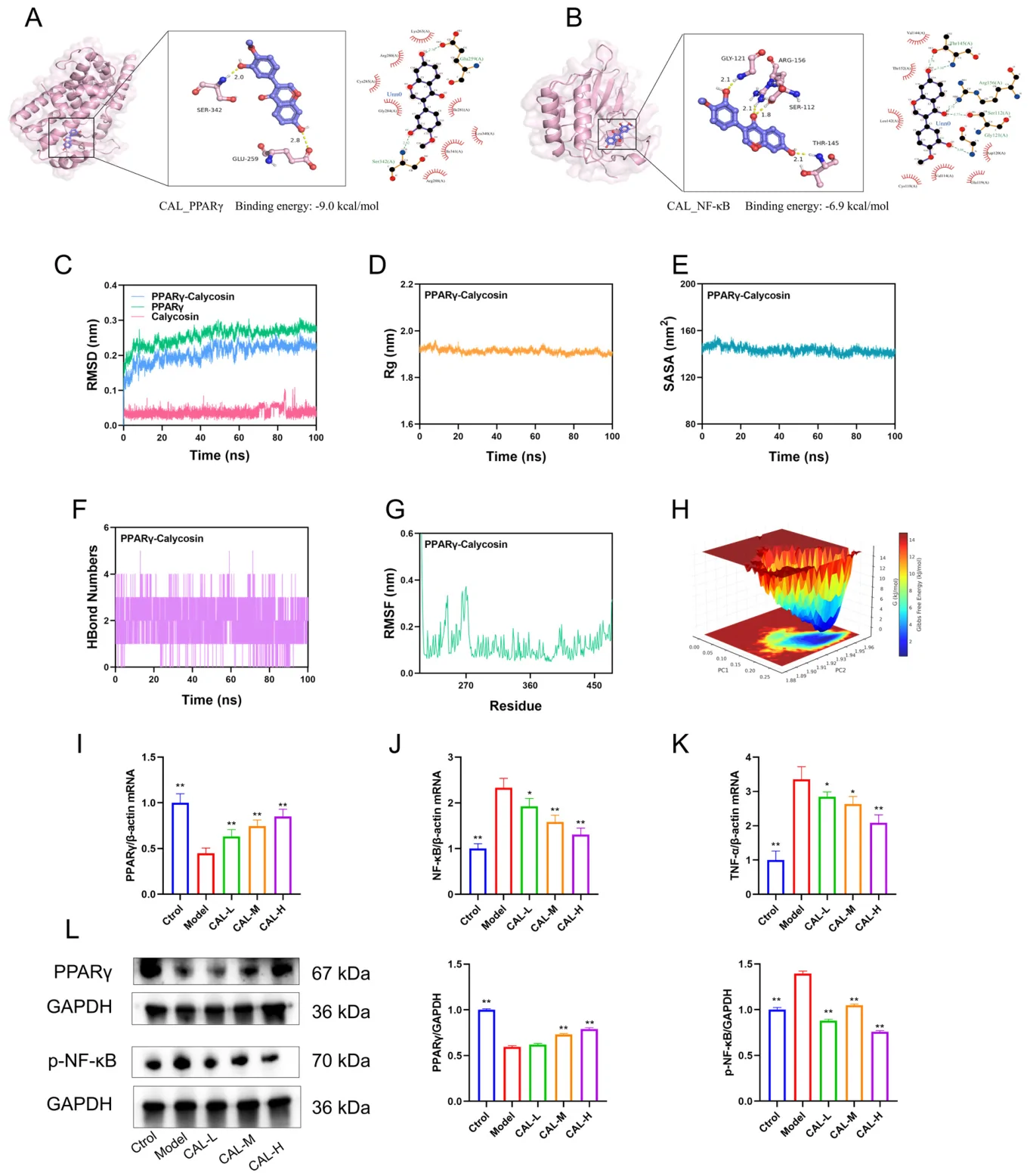
Effects of CAL on PPARγ/NF-κB signaling pathway in H9c2 cells. (**A**) Molecular docking of CAL with PPARγ. (**B**) Molecular docking of CAL with NF-κB. (**C**) Root-mean-square deviation (RMSD). (**D**) Radius of gyration (Rg). (**E**) Solvent-accessible surface area (SASA). (**F**) Hydrogen bond number. (**G**) Root-mean-square fluctuation (RMSF). (**H**) Free-energy landscape. (**I**–**K**) The relative mRNA expression levels of PPARγ, NF-κB, and TNF-α detected by RT-qPCR. (**L**) Western blot analysis of PPARγ and p-NF-κB. * *p* < 0.05 and ** *p* < 0.01 compared with Model group.

**Figure 7:**
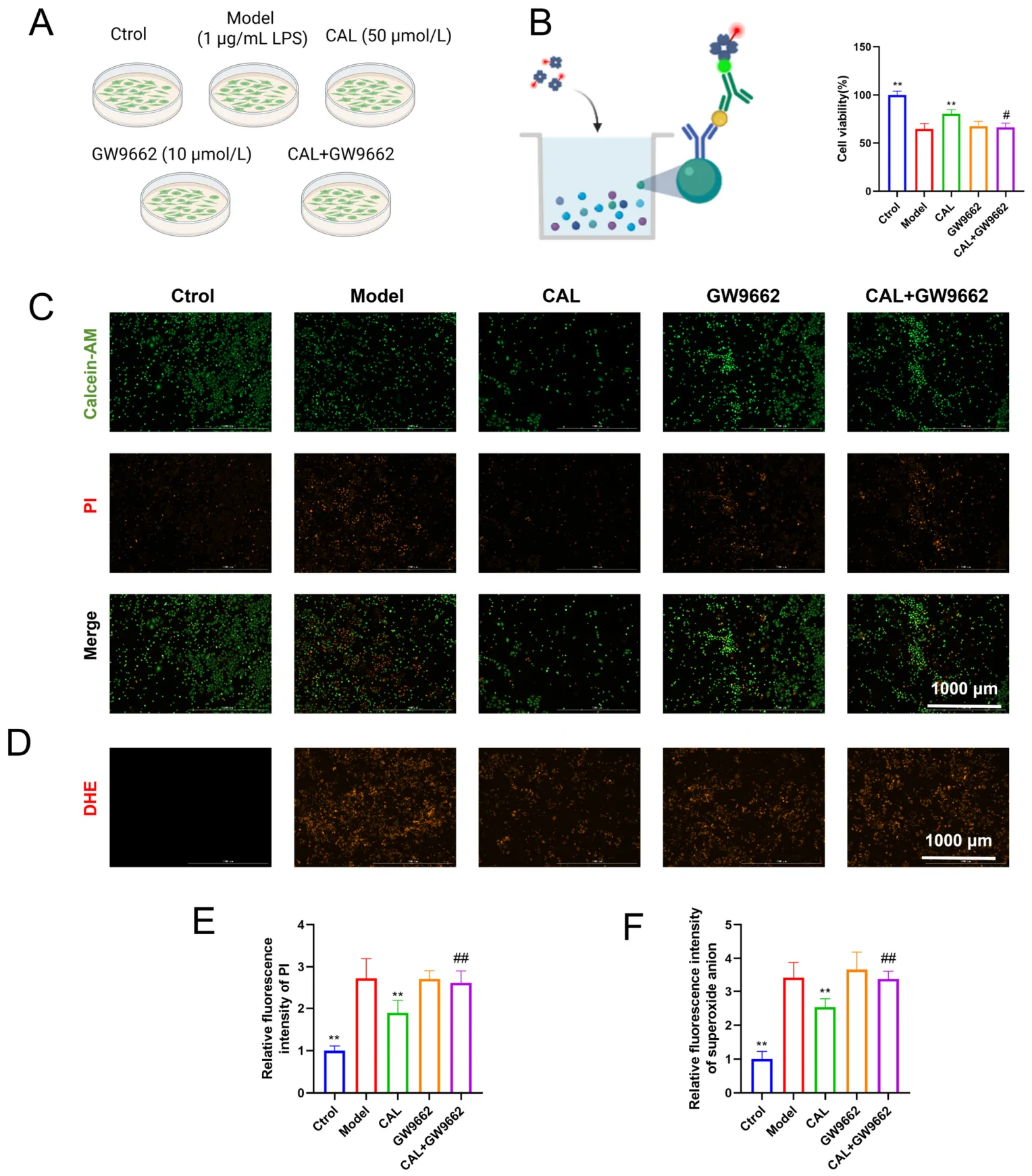
Effects of PPARγ inhibitor GW9662 on the protective function of CAL. (**A**) Schematic diagram of cell grouping and drug intervention; (**B**) cell viability detected by CCK-8 assay; (**C**) Calcein-AM/PI cell staining; (**D**) DHE staining; (**E**) relative fluorescence intensity of PI; (**F**) relative fluorescence intensity of superoxide anion. ** *p* < 0.01 compared with the Model group. ^#^ *p* < 0.05 and ^##^ *p* < 0.01 compared with the CAL group. Figure 7A,B were created with BioRender. Liang, K. (2026) https://BioRender.com/chm09me (accessed on 15 August 2026).

**Figure 8:**
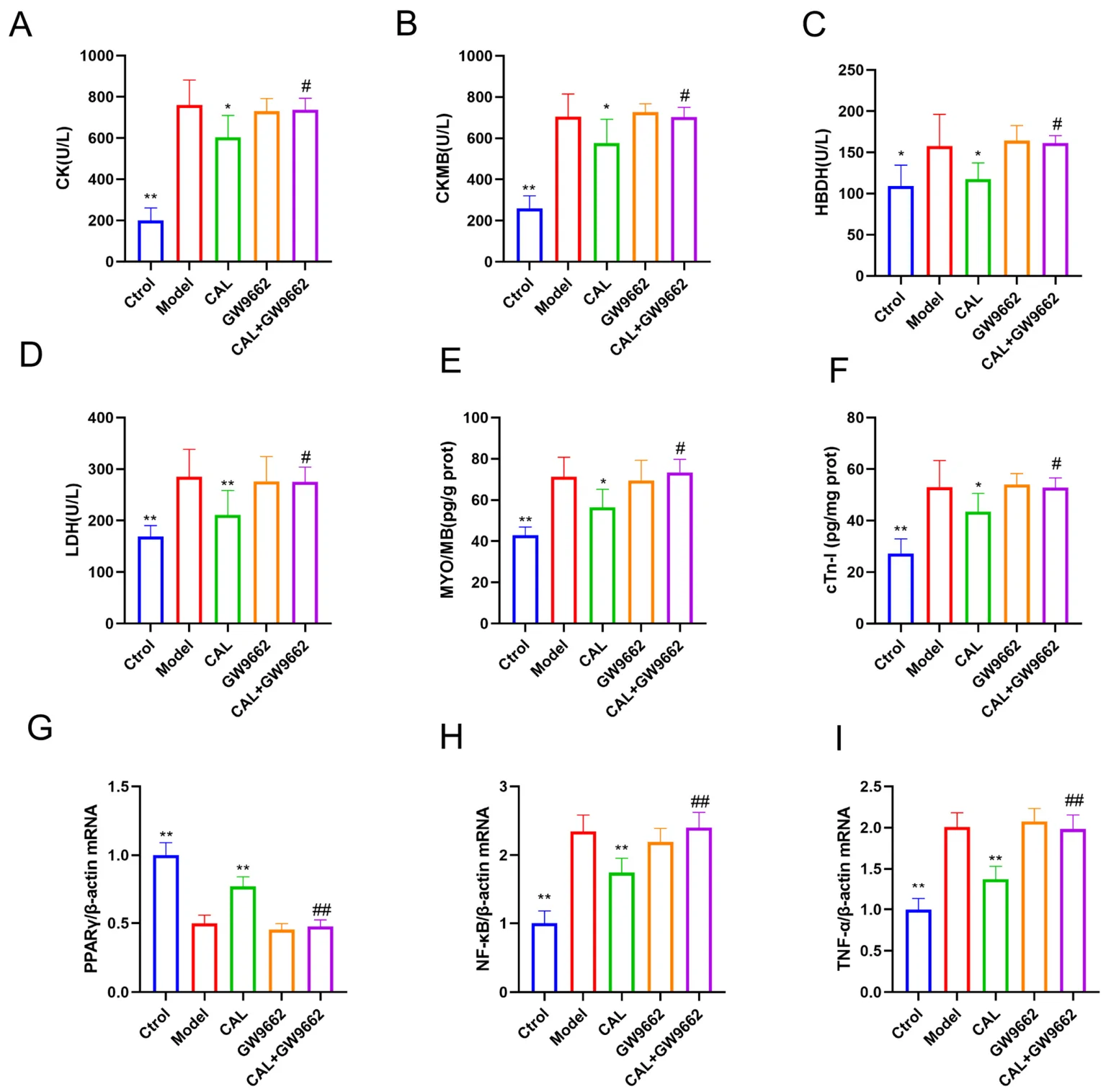
Effects of GW9662 on myocardial injury-related indicators and mRNA expression of PPARγ/NF-κB. (**A**) CK: creatine kinase; (**B**) CK-MB: creatine kinase-MB; (**C**) HBDH: α-hydroxybutyrate dehydrogenase; (**D**) LDH: lactate dehydrogenase; (**E**) MYO/MB: myoglobin; (**F**) cTn-I: cardiac troponin-I; (**G**) PPARγ: peroxisome proliferator-activated receptor-γ; (**H**) NF-κB: nuclear factor-κB; (**I**) TNF-α: tumor necrosis factor-α. * *p* < 0.05 and ** *p* < 0.01 compared with the Model group. ^#^ *p* < 0.05 and ^##^ *p* < 0.01 compared with the CAL group.

**Figure 9:**
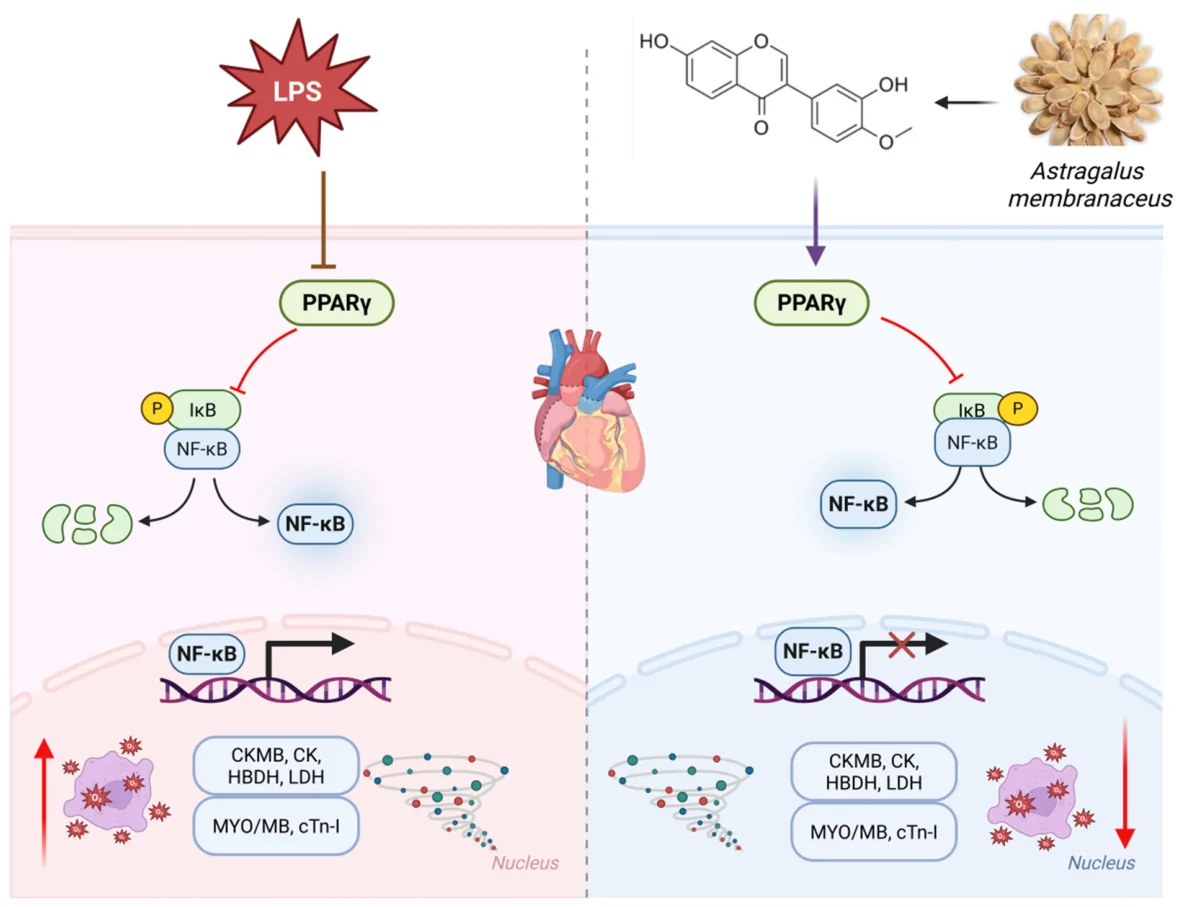
Schematic summary of the proposed mechanism by which CAL protects H9c2 cardiomyocytes against LPS-induced injury. CAL activates the PPARγ signaling pathway, which in turn suppresses NF-κB-mediated inflammatory responses and oxidative stress. This model is based on the present in vitro data and previously reported mechanisms, and should be regarded as a schematic summary rather than as a detailed mechanistic model. Created in BioRender. Liang, K. (2026) https://BioRender.com/chm09me (accessed on 15 August 2026).

**Table 1:** Primer sequences.

Name		Sequences (5′–3′)	Size (bp)
*PPARγ*	F	AAGGCTGCAGCGCTAAATTC	127
	R	GTGTCAACCATGGTAATTTCAGTA	
*NF-κB*	F	ACCGATGCATCCACAGCTTC	102
	R	AGGGGTTATTGTTGGTCTGGA	
*TNF-α*	F	GATCGGTCCCAACAAGGAGG	137
	R	CTTGGTGGTTTGCTACGACG	
*β-actin*	F	GGAGCATCCGACCTTGCTAA	169
	R	AGAGTCCAGCACAATACCAGT	

## Data Availability

The data used to support the findings of this study are available from the corresponding author upon request.

## Supplementary Materials

The following supporting information can be downloaded at https://www.mdpi.com/article/10.3390/ijms27198505/s1.
