# Supplementary material for: Calycosin Attenuates LPS-Induced Cardiomyocyte Injury Through Regulation of the PPARγ/NF-κB Signaling Pathway
Source: Int J Mol Sci. 2026 Sep 23;27(19):8505. doi: 10.3390/ijms27198505 (PMC13665561; doi:10.3390/ijms27198505)
Supplement: Supplementary file 1 [file ijms-27-08505-s001.zip › ijms-4562521-supplementary.pdf]

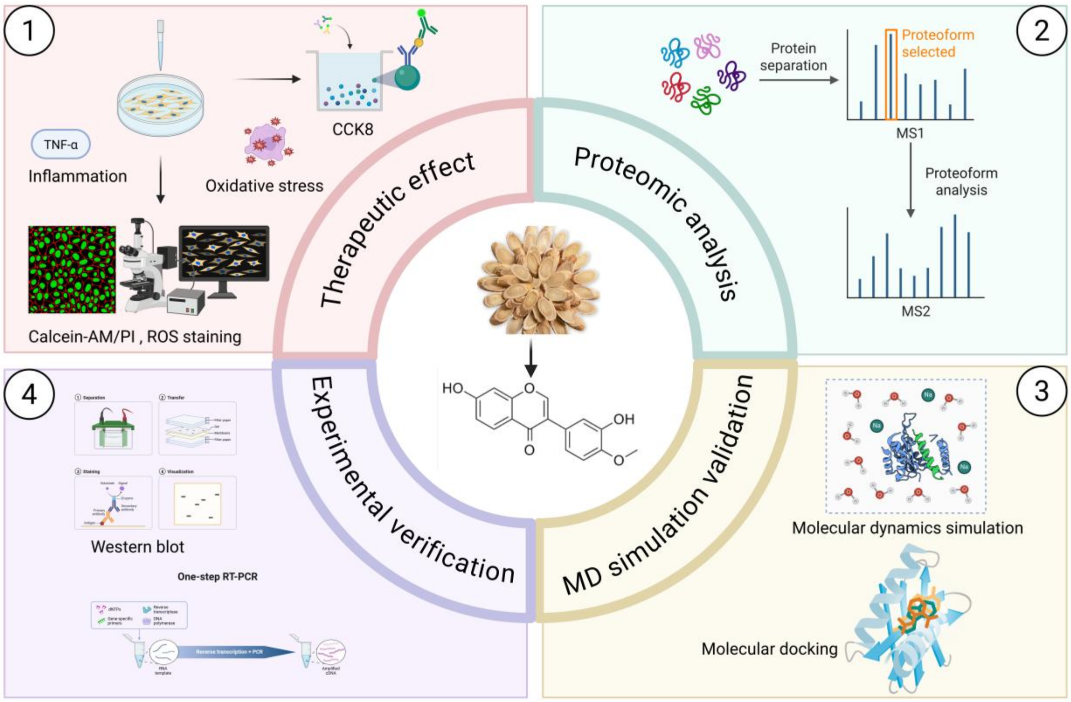

Figure S1 Integrative workflow of the study

1. Liquid chromatography

Chromatographic separation was performed on a Vanquish neo nano-UPLC system (Thermo Fisher Scientific, USA) coupled to an EASY-Spray™ HPLC reversed-phase column (150 μm × 15 cm, Thermo Fisher Scientific, USA) packed with 2 μm C18 particles. Mobile phase A was 0.1% formic acid in water, and mobile phase B was 0.1% formic acid in 80% acetonitrile. The flow rate was 300 nL/min, and the column temperature was maintained at 40 °C. Samples were loaded directly onto the column via an autosampler, with an injection volume corresponding to 1 μg of peptides. The complete LC gradient program is listed in Table S3.

Table S1. LC gradient program.

Time (min)	Mobile phase A (%)	Mobile phase B (%)
0	96	4
2	96	4
40	75	25
50	60	40
52	10	90

57	10	90
57.5	96	4
60	96	4

2. DIA window arrangement

The DIA method employed fixed, non-overlapping 2 m/z isolation windows spanning the 380–980 m/z precursor range, giving a total of 300 windows. Each 2 m/z window was sampled approximately every 1.67 s, yielding a median of 3 MS/MS data points per peak for a 15 min gradient and 5 MS/MS data points per peak for a 60 min gradient. MS1 spectra were acquired every 0.6 s, yielding a median of 15 MS1 data points per peak for a 60 min method.

3. Mass spectrometry acquisition parameters

Mass spectrometric data were acquired in data-independent acquisition (DIA) mode with positive ion detection. The full-scan (MS1) range was 380–980 m/z at a resolution of 240,000 (at m/z 200), with an AGC target of 3E6 and a maximum injection time of 50 ms. The isolation window was 2 m/z, and the normalized collision energy (NCE) was 25%. The MS2 scan range was 150-2000 m/z, with an absolute AGC value of 5E4 and a maximum injection time of 3 ms. MS2 spectra were acquired in the Astral mass analyzer, which provides MS/MS scan speeds exceeding 200 Hz. The duty cycle/cycle time was approximately 1.67 s. Four biological replicates were analysed per group.

4. Detailed DIA-NN data-processing and statistical workflow

4.1. Database search parameters

Raw DIA files were processed with DIA-NN v1.8.1 in library-free mode against the Rattus norvegicus UniProt reference proteome (UP000002494). The DIA-NN search parameters were as follows: enzyme specificity, Trypsin; allowed missed cleavages, 1; peptide length range, 7-30; precursor charge range, 1-4; fixed modification, carbamidomethylation of cysteine; variable modification, oxidation of methionine. The fragment ion m/z range was 100-1800. Match-between-runs (MBR)

was enabled. The false discovery rate (FDR) was controlled at <1% at both precursor and protein levels.

4.2. Protein quantification and normalization

Protein quantification was based on the MaxLFQ algorithm implemented in DIA-NN, with RT-dependent normalization applied across runs.

4.3. Missing value handling

Missing values were retained as missing and not imputed prior to differential testing. Only proteins quantified in at least 2 of 4 biological replicates in at least one group were retained for downstream analysis. Fold changes were calculated based on mean non-missing MaxLFQ intensities within each group. Proteins with fewer than 2 quantifications in both compared groups were excluded from that specific comparison.

5. Molecular docking parameters and binding-site residues

Table S2. Molecular docking parameters for CAL–PPARγ and CAL–NF-κB complexes.

Item	CAL–PPARγ	CAL–NF-κB
Receptor PDB ID	9f7w	8tqd
Receptor chain(s)	A	A, B
Predicted binding energy (kcal/mol)	−9.0	−6.9
Key pocket residues	Ser342, Glu259	Gly121, Arg156, Ser112, Thr145
Hydrogen-bonding residues and distances	Ser342: 2.0 Å; Glu259: 2.8 Å	Gly121: 2.1 Å; Arg156: 2.1 Å; Ser112: 1.8 Å; Thr145: 2.1 Å
Grid center (x, y, z) (Å)	46.459, 10.094, 17.958	13.14, 6.959, −0.607
Docking software	AutoDock Vina	AutoDock Vina
Exhaustiveness	8	8

Note on binding-site definition and docking protocol. For PPARγ, docking was targeted to the ligand-binding pocket of the ligand-binding domain, and the predicted

binding mode involved hydrogen bonds with Ser342 and Glu259 (Figure 6A). For NF-κB p65, docking was targeted to the region encompassing Gly121, Arg156, Ser112, and Thr145 (Figure 6B). Binding-site prediction was performed using AutoDockTools with manual grid box positioning based on the reported active-site residues. Redocking could not be performed because no suitable co-crystallized small-molecule ligand was available for the targeted pockets. The exhaustiveness parameter was set to 8, which represents the AutoDock Vina default and is recommended for reliable docking. AutoDock Vina scores are reported as predicted binding energies and do not constitute experimental evidence of binding.

Table S3. Summary of molecular dynamics simulations.

System	Atom selection	Equilibrated RMSD (nm)	Rg (nm)	SASA (nm^2)	H-bond number
PPARγ alone	PPARγ backbone	0.27	—	—	—
CAL alone	CAL heavy atoms	0.04	—	—	—
PPARγ–CAL complex	All atoms	0.25	1.93	148	2–4

Values are approximate means over the final 10–20 ns of each 100 ns trajectory. Rg and SASA were calculated for the PPARγ–CAL complex. Hydrogen-bond numbers refer to CAL–PPARγ interactions.
